# A New Oligonucleotide Microarray for Detection of Pathogenic and Non-Pathogenic *Legionella* spp.

**DOI:** 10.1371/journal.pone.0113863

**Published:** 2014-12-03

**Authors:** Boyang Cao, Xiangqian Liu, Xiang Yu, Min Chen, Lu Feng, Lei Wang

**Affiliations:** 1 Key Laboratory of Molecular Microbiology and Technology of the Ministry of Education, TEDA College, Nankai University, Tianjin, P. R. China; 2 TEDA School of Biological Sciences and Biotechnology, Nankai University, Tianjin, P. R. China; 3 Tianjin Research Center for Functional Genomics and Biochips, TEDA College, Nankai University, Tianjin, P. R. China; 4 Tianjin Key Laboratory of Microbial Functional Genomics, TEDA College, Nankai University, Tianjin, P. R. China; University of Louisville, United States of America

## Abstract

*Legionella pneumophila* has been recognized as the major cause of legionellosis since the discovery of the deadly disease. *Legionella* spp. other than *L. pneumophila* were later found to be responsible to many non-pneumophila infections. The non-*L. pneumophila* infections are likely under-detected because of a lack of effective diagnosis. In this report, we have sequenced the 16S-23S rRNA gene internal transcribed spacer (ITS) of 10 *Legionella* species and subspecies, including *L. anisa*, *L. bozemanii*, *L. dumoffii*, *L. fairfieldensis*, *L. gormanii*, *L. jordanis*, *L. maceachernii*, *L. micdadei*, *L. pneumophila* subspp. *fraseri* and *L. pneumophila* subspp. *pasculleii*, and developed a rapid oligonucleotide microarray detection technique accordingly to identify 12 most common *Legionella* spp., which consist of 11 pathogenic species of *L. anisa*, *L. bozemanii*, *L. dumoffii*, *L. gormanii*, *L. jordanis*, *L. longbeachae*, *L. maceachernii*, *L. micdadei*, and *L. pneumophila* (including subspp. *pneumophila*, subspp. *fraseri*, and subspp. *pasculleii*) and one non-pathogenic species, *L. fairfieldensis*. Twenty-nine probes that reproducibly detected multiple *Legionella* species with high specificity were included in the array. A total of 52 strains, including 30 target pathogens and 22 non-target bacteria, were used to verify the oligonucleotide microarray assay. The sensitivity of the detection was at 1.0 ng with genomic DNA or 13 CFU/100 mL with *Legionella* cultures. The microarray detected seven samples of air conditioner-condensed water with 100% accuracy, validating the technique as a promising method for applications in basic microbiology, clinical diagnosis, food safety, and epidemiological surveillance. The phylogenetic study based on the ITS has also revealed that the non-pathogenic *L. fairfieldensis* is the closest to *L. pneumophila* than the nine other pathogenic *Legionella* spp.

## Introduction

*Legionella* acquired its name after an outbreak of a then-unknown “mystery disease” that affected 221 persons, and caused 34 deaths eventually, attending a convention of the American Legion in July 1976. This epidemic, which occurred within days of the 200^th^ anniversary of the signing of the Declaration of Independence, was widely publicized and raised great concern in the United States [1]. A few months later, the causative agent was identified as a previously unknown bacterium, which was subsequently named *Legionella*. This gram-negative bacterium includes species responsible for Legionellosis or Legionnaire’s diseases, with *Legionella pneumophila* as the most notably species [2], [3]. Since then, more than 52 *Legionella* spp. have been identified (http://www.bacterio.cict.fr/l/legionella.html) [4], [5]. Although *L. pneumophila* remains as the major cause of legionellosis, non-pneumophila infections have been reported to be caused by *Legionella micdadei* (60%), *Legionella bozemanii* (15%), *Legionella dumoffii* (10%), *Legionella longbeachae* (5%), and other species (10%) [6].

Infections due to species other than *L. pneumophila* are likely to be underestimated because of a lack of appropriate diagnostic tests [6]. Since *Legionella* was first identified in 1977, various diagnostic tools for *Legionella* have been developed, including cell culture, antigen detection, serological typing, polymerase chain reaction (PCR), and microarray methods. The culture method is time-consuming due to the slow growth of *Legionella* spp., and it fails to distinguish *Legionella* spp. at the species level [7]. The detection of *Legionella* antigen in urine by enzyme immunoassays is a highly specific approach; and commercially available systems using this approach can detect *L. pneumophila* serogroup O1 but not other serogroups [8]. Serological typing methods with monoclonal and multiclonal antibodies can be used to detect *L. pneumophila* only with the aid of laborious pre-culture [9], [10].

Currently, most PCR methods target 5S rRNA, 16S rRNA, 23S-5S ribosomal RNA intergenic spacer, *mip*, *rpoB,* and *gyrB* genes [11]–[17]. However, the 5S, 16S, and 23S-5S rRNA genes are too conserved to differentially detect *L. pneumophila* from other *Legionella* spp. [18]. While the *mip* gene was initially used as an *L. pneumophila*-specific marker [19], other *Legionella* spp. were later found to harbor this gene as well [20], [21] A previous study has conducted a multilocus sequence analysis of 16S rRNA, *mip*, and *ropB* [22], and found 16S rRNA was useful for initial identification as it could recognize isolates robustly at the genus level, while *mip*, *rpoB*, and the *mip*-*rpo*B concatenation can be used to distinguish between different *Legionella* spp. However, multiplex PCR and sequencing are required for the identification, which render this method cumbersome and time-consuming. A *gyrB* gene-based single PCR method was developed for the differentiation of *L. pneumophila* subspp. *pneumophila* and *L. pneumophila* subspp. *fraseri*, but not for other *Legionella* spp. [16]. An oligonucleotide array based on *mip* gene sequences and digoxigenin-labeled PCR products was developed to identify 18 species of *Legionella* that have been reported to cause human infections, but the results are not reliable as some of the species only produced weak hybridization signals [13]. One other oligonucleotide microarray based on the *wzm* and *wzt* gene sequences and Cy3-labeled PCR products was developed to serotype all 15 distinct O-antigen forms within *L. pneumophila* [23].

In this study, we report the establishment of an oligonucleotide microarray method for the simultaneous detection of 11 pathogenic *Legionella* spp., *L. anisa, L. bozemanii, L. dumoffii, L. gormanii, L. jordanis, L. longbeachae, L. maceachernii, L. micdadei*, and *L. pneumophila* (including subspp. *pneumophila*, subspp. *fraseri*, and subspp. *pasculleii*), and one non-pathogenic spp., *L. fairfieldensis,* based on the 16-23S rRNA gene internal transcribed spacer (ITS) regions. The microarray method described here is specific, sensitive, and reliable and can be used as a better alternative to the traditional serotyping procedure, which is laborious and frequently cross-reactive.

## Materials and Methods

### Bacterial strains

The following standard *Legionella* spp. strains were used for ITS sequencing: *L. anisa* (DSMZ 17627), *L. bozemanii* (ATCC 33217), *L. dumoffii* (ATCC 33279), *L. fairfieldensis* (ATCC 49588), *L. gormanii* (ATCC 43769), *L. jordanis* (DSMZ 19212), *L. maceachernii* (DSMZ 16642), *L. micdadei* (NCTC 11371), *L. pneumophila* subspp. *fraseri* (ATCC 35251), and *L. pneumophila* subspp. *pascullei* (ATCC 4585). The 52 bacterial strains used for microarray are listed and described in Table 1, and included 30 strains of the *Legionella* target species and 22 other non-target bacterial species. Of these 52 strains, 41 were reference strains and 11 were clinical or environmental isolates. *Legionella* strains were cultured onto buffered charcoal yeast extract (BCYE) agar plates (Hope Bio-technology Co., Ltd, Qingdao, China) and incubated in a 5% CO_2_ incubator at 37°C for 2–4 days.

**Table 1:** Bacterial strains used in this study.

Bacterium	No. of strains of each source	Total number
**Target ** ***Legionella*** ** spp. used to test the specificity of the probes (n = 30)**		
*Legionella pneumophila* (subspp. *pneumophila*)	1^a^, 10^b^, 2^c^, 1 k	14
*Legionella pneumophila* (subspp. *fraseri)*	1^c^, 1^d^, 1^k^	3
*Legionella pneumophila* (subspp. *pascullei*)	1^c^	1
*Legionella anisa*	1^d^	1
*Legionella bozemanii*	1^c^, 1^j^	2
*Legionella dumoffii*	1^c^	1
*Legionella fairfieldensis*	1^c^	1
*Legionella gormanii*	1^c^	1
*Legionella jordanis*	1^d^	1
*Legionella longbeachae*	1^d^, 1^j^	2
*Legionella maceachernii*	1^d^	1
*Legionella micdadei*	1^b^, 1^j^	2
**Other bacterial species used to test the specificity of the probes (n = 22)**		
*Legionella feeleii*	1^j^	1
*Legionella steigerwaltii*	1^b^	1
*Legionella worsleiensis*	1^c^	1
*Acinetobacter baumannii*	1^g^	1
*Citrobacter freundii*	1^f^	1
*Escherichia coli*	1^f^	1
*Enterococcus faecalis*	1^e^	1
*Klebsiella pneumoniae*	1^f^	1
*Listeria monocytogenes*	1^h^	1
*Proteus mirabilis*	1^i^	1
*Proteus penneri*	1^a^	1
*Proteus vulgaris*	1^i^	1
*Pseudomonas aeruginosa*	1^e^	1
*Salmonella paratyphi*	1^f^	1
*Salmonella typhi*	1^f^	1
*Shigella boydii*	1^k^	1
*Shigella flexneri*	1^c^	1
*Streptococcus pneumoniae*	1^c^	1
*Streptococcus pyogenes*	1^f^	1
*Staphylococcus aureus*	1^e^	1
*Staphylococcus epidermidis*	1^f^	1
*Vibrio parahaemolyticus*	1^j^	1
**Bacterial species used to perform the blind test (n = 22)**		
*Legionella pneumophila* (subspp. *pneumophila*)	1^a^	1
*Legionella pneumophila* (subspp. *fraseri*)	1^d^	1
*Legionella pneumophila* (subspp. *pascullei*)	1^c^	1
*Legionella anisa*	1^d^	1
*Legionella bozemanii*	1^c^	1
*Legionella dumoffii*	1^c^	1
*Legionella fairfieldensis*	1^c^	1
*Legionella gormanii*	1^c^	1
*Legionella jordanis*	1^d^	1
*Legionella longbeachae*	1^d^	1
*Legionella maceachernii*	1^d^	1
*Legionella micdadei*	1^b^	1
*Legionella steigerwaltii*	1^b^	1
*Legionella worsleiensis*	1^c^	1
*Escherichia coli*	1^f^	1
*Enterococcus faecalis*	1^e^	1
*Klebsiella pneumoniae*	1^f^	1
*Pseudomonas aeruginosa*	1^e^	1
*Salmonella typhi*	1^f^	1
*Staphylococcus aureus*	1^e^	1
*Streptococcus pneumoniae*	1^c^	1

a Czech Collection of Microorganisms (CCM), Masaryk University, Brno, Czech Republic.

b National Collection of Type Cultures (NCTC), Central Public Health Laboratory, London, United Kingdom.

c American Type Culture Collection (ATCC), USA.

d German Collection of Microorganisms and Cell Cultures (DSMZ), Germany.

e Institute of Microbiology, Chinese Academy of Sciences (IMCAS).

f National Center for Medical Culture Collections (CMCC), Beijing, China.

g Universityät zu Köln, Deutschland, Gernamy.

h Agricultural Culture Collection of China (ACCC), Beijing, China.

i University of Lodz, Poland.

j Shanghai Municipal Center for Disease Control and Prevention.

k Shenzhen NanShan Center for Disease Control and Prevention.

### Genomic DNA preparation

Genomic DNA was extracted from pure cultures using bacterial genomic DNA purification kit (Tiangen Biotech Co., Ltd., Beijing, China).

### Amplification of *Legionella* spp. ITS regions

The primer pair wl-5793 and wl-5794 was designed on the basis of the 16S rRNA gene and 23S rRNA gene sequences, respectively, using Primer Premier 5.0 software (Premier Boost International, CA) [24], [25]. These primers were used to amplify the ITS region of all *Legionella* spp. The primer sequences and concentrations used for multiplex PCR are listed in Table 2. The PCR mixture contained 1× PCR buffer (50 mM KCl, 10 mM Tris-HCl; pH 8.3), 2.5 mM MgCl_2_, 200 µM dNTP, 1.0 U *Taq* DNA polymerase, 10 nM of each of the primers, and 100 ng of DNA template in a final volume of 50 µl. PCR was performed by initial denaturation at 95°C for 5 min; followed by 30 cycles of 94°C for 30 s, 50°C for 30 s, and 72°C for 1 min; and a final extension at 72°C for 5 min. The amplified fragment was then checked by agarose gel electrophoresis of 2-µL aliquots of the PCR products.

**Table 2:** Primers and their concentrations in multiplex PCR.

Primer name	Target gene	Sequence (5′–3′)^a^	PCR product size (bp)	GenBank accession no.	Primer Conc. in multiplex PCR (µM)	Primer Conc. for Labelling (µM)
wl-5793	ITS	(F)TGTACACACCGCCCGTC	500–1000	CP000675.2	0.2	
wl-5794		(R)GGTACTTAGATGTTTCAGTTC			0.2	0.2

a F, forward primer; R, reverse primer.

### Cloning and sequencing of the ITS regions of *Legionella* spp. and subspp

PCR amplicons were cloned into the pGEM-T Easy vector (Promega, MA) and transformed into *E. coli* DH5α. Transformants (observed as white colonies grown on an ampicillin plates containing isopropyl-beta-d-thiogalactopyranoside and X-gal) were selected randomly. Plasmid DNA was isolated using the conventional alkaline lysis method, digested with *Eco* RI, and visualized on agarose gels to confirm the presence of the corresponding inserts. Sequences were verified using an ABI 3730 automated DNA sequencer (Applied Biosystems, USA). Sixteen transformants per *Legionella* spp. or subspp. were examined.

### Sequence analysis

Multiple sequence alignment of ITS sequences was carried out with the ClustalW program (http://www.ebi.ac.uk/clustalw/). The identity level was calculated using BioEdit software (http://www.mbio.ncsu.edu/BioEdit/page2.html). Phylogenetic trees were constructed using the neighbor-joining method and plotted by the molecular evolutionary genetics analysis (MEGA) 3.1 software package (http://www.megasoftware.net). Bootstrap analysis was carried out based on 1,000 replicates.

### Target DNA amplification and labeling

Primer concentrations were optimized according to the final intensity of the microarray hybridization signals. The PCR mixtures contained 1×PCR buffer (50 mM KCl, 10 mM Tris-HCl; pH 8.3), 2.5 mM MgCl_2_, 400 µM dNTP, 0.2 µM ITS of each primer, 2.5 U *Taq* DNA polymerase, and 50–100 ng of DNA template in a final volume of 25 µL. The following PCR parameters were employed: initial denaturation at 95°C for 5 min; followed by 35 cycles of 95°C for 30 s, 50°C for 30 s, and 72°C for 1 min; and a final extension at 72°C for 5 min. The amplified DNA was analyzed by agarose gel electrophoresis of a 2-µL aliquot of the PCR product (Fig. S1). To label the PCR products, 10 µL of the PCR products generated from the first run and the reverse primer and 0.3 µL of 25 nM Cy3-dUTP were added to the PCR mixture, and PCR was carried out using the same PCR conditions described above.

### Oligonucleotide probe design

The conserved and variable regions of the ITS sequences were defined by aligning multiple ITS sequences using ClustalW. For each type of pathogen, two to four probes were designed on MEGA 3.1 based on the sequences from the GenBank database or from our lab data, and checked by Primer Premier 5.0. One probe based on the 16S rRNA gene was designed as the positive control (OA-1993). A probe containing 40 poly(T) oligonucleotides was used as the negative control (WL-4006). A probe containing 40 poly(T) oligonucleotides labeled by 3′-Cy3 was used as the positional reference and printing control (Cy3). Each probe comprised a modified 5′ amino acid sequence followed by a spacer of 10 to 15 poly(T)s and a stretch of specific sequence (synthesized by AuGCT Biotechnology Corporation, Beijing, China). All the oligonucleotide probes used in this study are listed in Table 3.

**Table 3:** Oligonucleotide probes used in this study.

Target species	Probe name	T_m_ (°C)^a^	Sequence (5′–3′)	GenBank accession no.
*Legionella anisa*	OA-3819	68.5	GCATGCATCAGTATGTGACCAAGCGAGCGAG	KM609989
	OA-3820	70.1	CGAGCGAGTGGATGCAATGAAAACAAATTT	
*Legionella bozemanii*	OA-3821	68.5	AAAGCCGTGACCGAGAGGAAGCGGGAAGA	KM609990
	OA-3822	78.6	AAGCGGGAAGATGCGCGGTCACGCTGAAAGC	
	OA-3823	73.8	TCGTGACCGAGAGGAAGCGGGAAGATGCGC	
*Legionella dumoffii*	OA-3824	57.2	TGAATGATGAATAAATCCTAAGCTTCTGAA	KM609992 KM609993
	OA-3825	62.4	CTGAAAGGAAGCAAATGCTTGATAAAGC	
	OA-3826	63.8	TCCTAACCGTAATTTTTTATGCGGAAAGAAT	
*Legionella fairfieldensis*	OA-3827	66.6	GATTGCCGTATTTTTTGGGTGGATTGGAATG	KM609994
	OA-3828	60.1	AGAGTCTGCATTGTGTAGCATTGATTATTG	
	OA-3829	69.1	TGGGTGGATTGGAATGGTTTCATGAA	
*Legionella gormanii*	OA-3830	69.2	TGAGCCCGGTTCATAACGTTGTGAGTGCGGC	KM609996 KM609997
	OA-3831	50.4	AGATAATTTTTCTTTAGTTCAAGTAAGTGTT	
	OA-3832	65.2	GTAAAATTGCACTGTCTTGCGTTGGAG	
*Legionella jordansis*	OA-3833	77.3	ACTCCGATGCGAGGGAGCGAAGCGACCAA	KM609999
	OA-3834	71.8	TGCTGAGCGAGGGAGCTTCGTAACCAAGGGGT	
	OA-3835	62.4	ACCTTTATTGATTTTAGCGATGGCTTTGAA	
*Legionella longbeachae*	OA-3836	63.9	AAGCGGTAACAAAAGAGTGACTCGAAGC	NC_013861
	OA-3837	60.5	CCAATTTTAGGGTTTTCAAGGATAGTCCA	
	OA-3838	57.5	CAGAAAGATGAAAAATCTTAAGCTGCG	
*Legionella micdadei*	OA-3839	65.9	ATTCCTTAATCGAGATGTCAACGCGAAGG	
	OA-3840	69.3	GCTCGGTATGTGACCGAGGAAAAGCATGTC	
	OA-3841	75.7	TACCGATGGCGCTTGGAACGGGCTAATGAGCC	
*Legionella maceachernii*	OA-3842	57.9	AAGACAAGGAAAAGAATAGCAGATTCTGCG	
	OA-3843	69.4	GCATCGGTGCGAAAGAGTAAGGGAGCCTACG	
	OA-3844	68.0	ACGCAAGTAGGTGGCTGAACGAAAGGGTAT	
*Legionella pneumophila* (subspp *pneumophila,*subspp *fraseri,* subspp *pascullei*)	OA-3815	71.3	CAAGAATCGGAACGCGGTCCAAGATTGG	CP000675.2
	OA-3816	65.3	AAGCGATTGGTATTTGCATCATGTGATTT	
	OA-3817	59.2	CATAGAAAGGCACAGAAGGAACTAGAGTGC	
Positive control	OA-1993	68.5	GTACACACCGCCCGTCACACCAT^b^	X80725
Negative control	WL-4006		TTTTTTTTTTTTTTTTTTTTTTTTTTTTTTTTTTTTTTTT^c^	
Positional reference & printing control	Cy3		TTTTTTTTTTTTTTTTTTTTTTTTTTTTTTTTTTTTTTTT _Cy3^d^	

a Tm was predicted using Primer Premier 5.0 software.

b The 16S rDNA based probe was used as the positive control.

c The probe containing 40 poly(T) oligonucleotides was used as the negative control.

d The probe labeled by 3′-Cy3 was used as the positional reference and printing control.

### Microarray preparation

**Figure 1:**
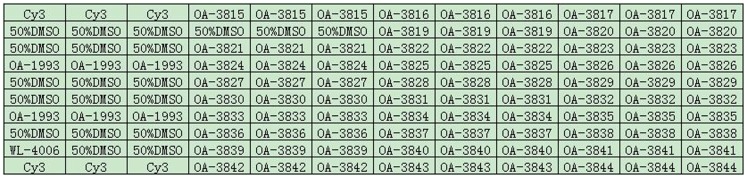
Probe positions on the microarray. OA-1993, the positive control probe based on the 16S rRNA gene. WL-4006, the negative control probe. Cy3, the positional reference and printing control probe. Blank, 50%DMSO. The rest are the specific probes for the target strains.

The probes were dissolved in 50% dimethyl sulfoxide (DMSO) to a final concentration of 1 µg/µL and coated onto aldehyde group-modified glass slides (CapitalBio Corporation, Beijing, China) using SpotArray 72 (Perkin-Elmer Corporation, CA, USA). Each probe was spotted in triplicate. Coated slides were dried and stored at room temperature in the dark. Each glass slide contained eight individual arrays framed with an 8-sample cover slip containing individual reaction chambers. A schematic diagram of the probe positions on the microarray is shown in Fig. 1.

### Microarray hybridization and data analysis

All labeled PCR products were precipitated using 100% cold ethanol, centrifuged at 13,000 *g* for 10 min, washed with 75% ethanol, and dried at room temperature. The dried, labeled DNA was diluted in 16 µL of hybridization buffer (50% formamide, 6× SSC, 5× Denhardt, and 0.5% SDS) and then hybridized with the prepared microarray at 45°C for 12 h. After hybridization, the slide was washed with solution A (1× SSC and 0.1% SDS) for 3 min, solution B (0.05×SSC) for 3 min, and solution C (95% ethanol) for 1.5 min. The microarray was dried under a gentle air stream and scanned with a laser beam of 532 nm using the GenePix biochip scanner 4100A (Axon Instruments, CA, USA) set to the following parameters: photomultiplier tube gain, 600, and pixel size, 5 µm. The signal-to-noise ratio (SNR) was calculated for each spot using the built-in software, GenePix Pro 6.0, with the threshold set at 3.0. A signal was considered positive when 70% of the probes to a respective target gene generated hybridization signals above the SNR threshold.

### Test of mock samples

BCYE medium was used for proliferation. Pure cultures of *L. bozemanii*, *L. dumoffii*, and *L. gormanii* were serially diluted from 10^1^ to 10^6^ CFU/mL, and 1 mL of the diluent was mixed with 100 mL of fresh tap water from the laboratory and vacuum filtered through a 0.22-µm membrane. The membrane was treated with 500 µL of diluted HCl (pH 3.0) for 1 min, placed face-down on BCYE agar plates, and incubated in a 5% CO_2_ incubator at 37°C for 3–5 days. The genomic DNA was then extracted from the cells for microarray hybridization.

### Test of air conditioner-condensed water samples

A filter-enriched air conditioner-condensed water sample (800 µL) was plated onto a GVPC agar plate (Hope Bio-Technology Co., Ltd., Qingdao, China) and incubated in a 5% CO_2_ incubator at 37°C for 48 h. Then, the culture was collected and genomic DNA was extracted for use in the downstream PCR and hybridization process.

### Nucleotide sequence and microarray accession numbers

The ITS sequences of *L. anisa, L. bozemanii, L. dumoffii, L. fairfieldensis, L. gormanii, L. jordanis, L. maceachernii, L. micdadei*, and *L. pneumophila* subspp. *fraseri*, and *L. pneumophila* subspp. *pascullei* were deposited into the GenBank database under the accession numbers KM609984-KM610004. The microarray dataset was deposited into the Gene Expression Omnibus database under the accession number GSE61962.

## Results

### *Legionella* spp. ITS regions reveal interspecies variations

We have sequenced the ITS regions of 10 *Legionella* spp. and subspp., including *L. anisa, L. bozemanii, L. dumoffii, L. fairfieldensis, L. gormanii, L. jordanis, L. maceachernii, L. micdadei, L. pneumophila* subspp. *fraseri* and *L. pneumophila* subspp. *pasculleii*. Next we analyzed the ITS regions of the 12 *Legionella* spp., the above 10 plus *L. longbeachae* and *L. pneumophila* subspp. *pneumophila*, whose sequences were previously published (NC_013861, CP0005672) using tRNA-ScanE software (http://lowelab.ucsc.edu/tRNAscan-SE/). The data indicated that except for *L. jordanis*, which has three ITS types of ITS-tRNA^Ala^ (with tRNA^Ala^ gene), ITS-tRNA^Ile^ (with tRNA^Ile^ gene), and ITS-tRNA^none^ (without tRNA gene), the 11 other *Legionella* spp. and subspp. all contain two distinct ITS types: ITS-tRNA^Ala^ (with tRNA^Ala^ gene) and ITS-tRNA^Ile^ (with tRNA^Ile^ gene). Alignments of the above ITS sequences revealed significant interspecies variations of 0.266–0.772 for ITS-tRNA^Ala^ and 0.280-0.774 for ITS-tRNA^Ile^, but low intraspecies polymorphisms of 0.967–0.993 for *L. pneumophila* ITS-tRNA^Ala^ and 0.943–0.996 for *L. pneumophila* ITS-tRNAI^le^, suggesting that ITS is a good target for species-specific identification.

### Phylogenetic analysis

**Figure 2:**
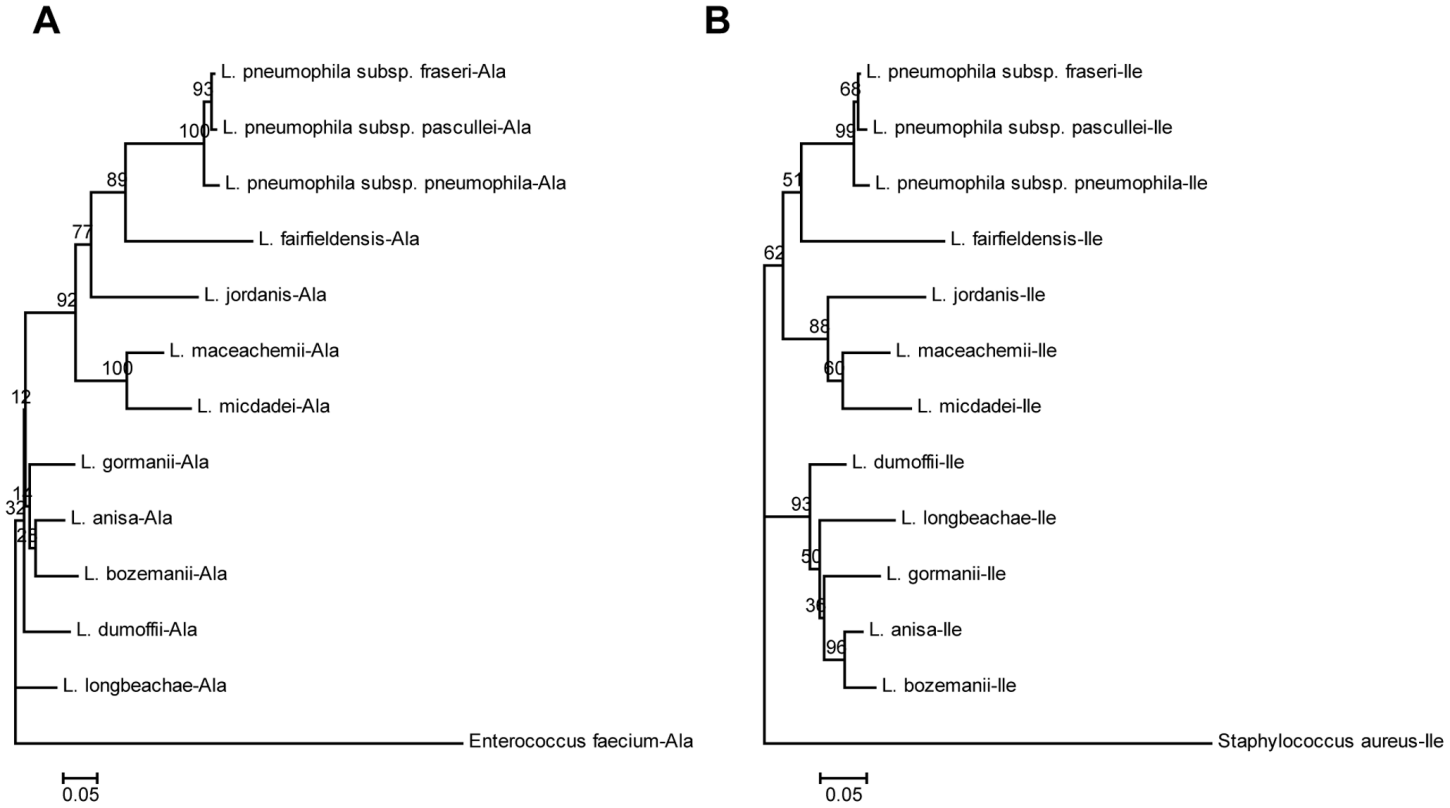
Unrooted phylogenetic trees constructed by the neighbor-joining method based on the ITS-tRNA^Ala^ and ITS-tRNA^Ile^ genes. Bootstrap values were based on 1,000 replications and only values greater than 50% are shown. A, Unrooted ITS-tRNA^Ala^ gene phylogenetic tree of *Legionella* spp. and subspp. constructed with the neighbor-joining method. B, Unrooted ITS-tRNA^Ile^ gene phylogenetic tree of *Legionella* spp. and subspp. constructed with the neighbor-joining method.

We have constructed two phylogenetic trees of 12 *Legionella* spp. and subspp. based on the ITS-tRNA^Ala^ and ITS-tRNA^Ile^ gene sequences with *Staphylococcus aureus* and *Enterococcus faecium* as the outer group references for the two gene sequences (Fig. 2A and 2B). In the ITS-tRNA^Ala^ tree, there are two subgroups: the first subgroup consists of *L. pneumophila* subspp. *pneumophila*, *L. pneumophila* subspp. *fraseri*, *L. pneumophila* subspp. *pascullei, L. fairfieldensis*, *L. jordanis*, *L. maceachernii*, and *L micdadei;* while the second contained subgroup, *L. gormanii*, *L. anisa*, *L. bozemanii*, *L. dumoffii*, and *L. longbeachae*. In the ITS-tRNA^Ile^ tree, there are three subgroups: the first subgroup includes *L. pneumophila* subspp. *pneumophila*, *L. pneumophila* subspp. *fraseri*, *L. pneumophila* subspp. *pascullei* and *L. fairfieldensis*; the second subgroup, *L. jordanis*, *L. maceachernii*, and *L micdadei;* the third subgroup, *L. dumoffii*, *L. longbeachae*, *L. gormanii*, *L. anisa*, and *L. bozemanii*. In both phylogenetic trees, *L. pneumophila* is found to be most closely related to *L. fairfieldensis*; *L. Maceachernii* is closest to *L micdadei*; and *L. jordanis* is in the neighborhood of *L. fairfieldensis* and *L. maceachernii;* and *L. anisa* is closest to *L. bozemanii*.

### Probe specificity

A total of 52 strains were used to test the specificity of the designed probes. Probes that cross-hybridized or did not produce signals were eliminated from the test panel. After the screening, 32 probes (including 29 species-specific probes, one positive control probe, one negative control probe, and one positional and printing control probe) were selected (Table 3).

**Figure 3:**
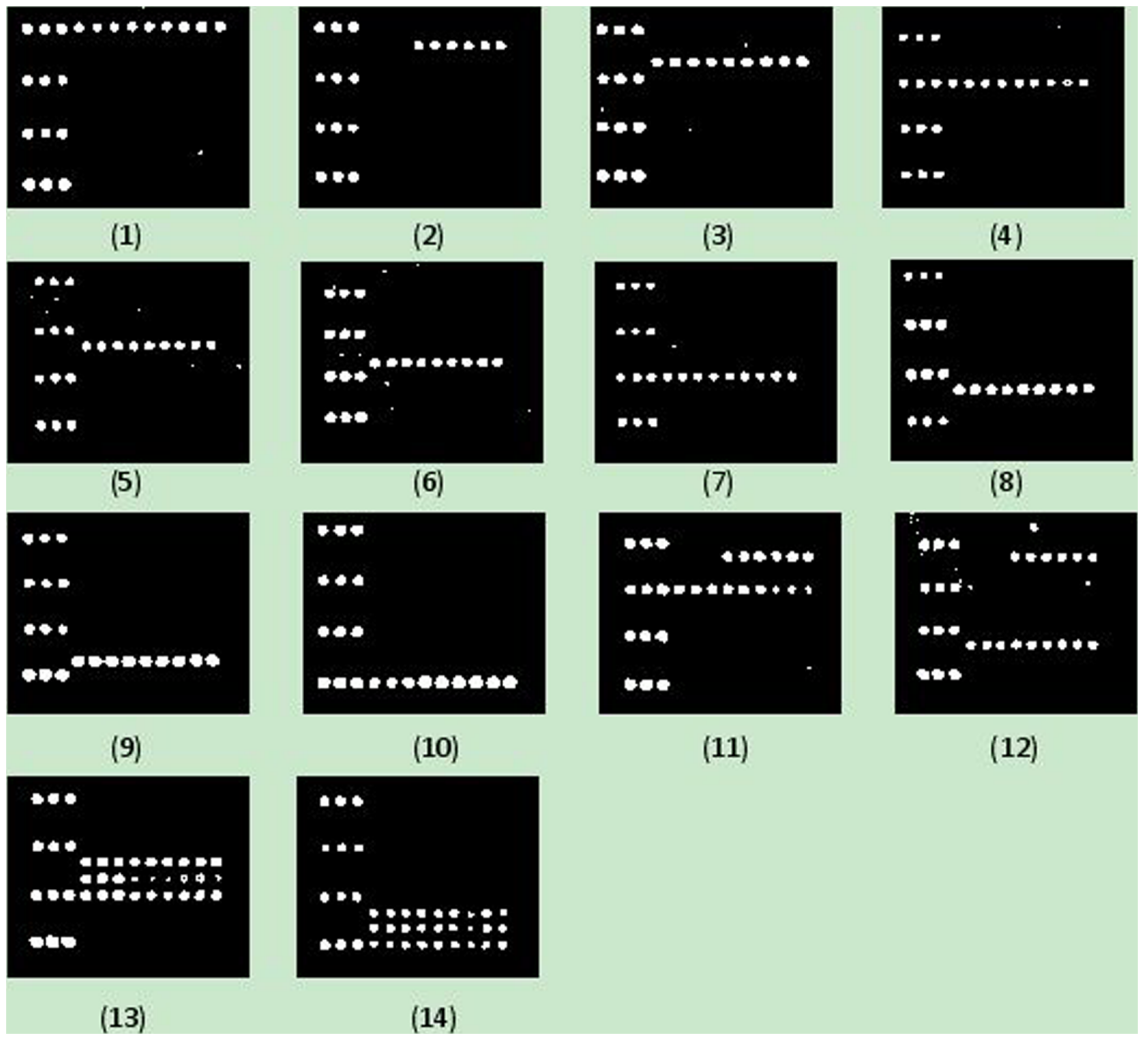
Microarray differentiation of the *Legionella* spp. *L. pneumophila*; (2) *L. anisa*; (3) *L. bozemanii*; (4) *L. dumoffii*; (5) *L. fairfieldensis*; (6) *L. gormanii*; (7) *L. jordanis*; (8) *L. longbeachae*; (9) *L. micdadei*; (10) *L. maceachernii*; (11) *L. anisa* and *L. dumoffii*; (12) *L. anisa* and *L. longbeachae*; (13) *L. fairfieldensis*, *L. gormanii* and *L. jordanis*; and (14) *L. longbeachae*, *L. maceachernii* and *L. micdadei*.

The microarray specifically identified the 30 target strains. For example, *L. pneumophila* (including subspp. *pneumophila*, subspp. *fraseri*, and subspp. *pasculleii*) produced positive signals with its specific probes of OA-3815, OA-3816, and OA-3817, as well as the positive control probe OA-1993 and the positional and printing control probe Cy3 but not with the other probes [Fig. 3(1)]. Likewise, *L. anisa* produced positive signals with its specific probes of OA-3819 and OA-3820 [Fig. 3(2)]; *L. bozemanii*, with OA-3821, OA-3822, and OA-3823 [Fig. 3(3)]; *L. dumoffii*, with OA-3824, OA-3825, and OA-3826 [Fig. 3(4)]; *L. fairfieldensis*, with OA-3827, OA-3828, and OA-3829 [Fig. 3(5)]; *L. gormanii*, with OA-3830, OA-3831, and OA-3832 [Fig. 3(6)]; *L. jordanis*, with OA-3833, OA-3834, and OA-3835 [Fig. 3(7)]; *L. longbeachae*, with OA-3836, OA-3837, and OA-3838 [Fig. 3(8)]; *L. maceachernii*, with OA-3839, OA-3840, and OA-3841 [Fig. 3(9)]; *L. micdadei*, with OA-3839, OA-3840, and OA-3841 [Fig. 3(10)]. At the same time, none of the 22 non-target bacteria produced positive signals with the 29 *Legionella* spp. specific probes, demonstrating that the designed probes were species-specific.

### Microarray sensitivity

The sensitivity of the microarray analysis was tested by hybridization with serially diluted genomic template DNA at concentrations of 0.1, 1.0, 10, and 100 ng. Based on the positive signals generated, the sensitivity of the assay using genomic DNA was 1.0 ng DNA for *L. pneumophila*, *L. longbeachae,* and *L micdadei*, and 0.1 ng DNA for *L. dumoffii* (Fig. S2).

### Simultaneous detection of multiple pathogens

As the detection will be more desirable if multiple pathogens can be simultaneously detected. Genomic DNA of two groups of two pathogens: *L. anisa* and *L. dumoffii* or *L. anisa* and *L. longbeachae* were mixed and used as templates for the testing. The results revealed that the probes were able to hybridize specificly the target regions of these pathogens, demonstrating the designed probes are able to detect multiple samples simultaneously [Fig. 3(11)-3(12)]. Next, genomic DNA of two groups of three pathogens: *L. gormanii*, *L. jordanis*, and *L. fairfieldensis* or *L. maceachernii*, *L micdadei*, and *L. longbeachae*, were mixed and again the microarray probes were able to successfully identify multiple pathogens simultaneously [Fig. 3(13) and 3(14)].

### Blind test

The specificity and sensitivity of the microarray detection system described was blind tested. Coded DNA samples from 22 species (Table 1) were randomly selected and hybridized to the microarrays. The bacterial species selected were *L. anisa* (n = 1), *L. bozemanii* (n = 1), *L. dumoffii* (n = 1), *L. fairfieldensis* (n = 1), *L. gormanii* (n = 1), *L. jordanis* (n = 1), *L. longbeachae* (n = 1), *L. maceachernii* (n = 1), *L. micdadei* (n = 1), *L. pneumophila* (n = 3), *Legionella steigerwaltii* (n = 1), *Legionella worsleiensis* (n = 1), *Escherichia coli* (n = 1), *Enterococcus faecalis* (n = 1), *Klebsiella pneumoniae* (n = 1), *Pseudomonas aeruginosa* (n = 1), *Salmonella typhi* (n = 1), *Staphylococcus aureus* (n = 1), and *Streptococcus pneumoniae* (n = 1). The results matched exactly with that of the conventional detection methods (data not shown).

### Test of mock samples

The mock samples containing *L. bozemanii*, *L. dumoffii*, and *L. gormanii* at various concentrations were tested, and the detection level was found to be at 18, 15, and 5 CFU/100 mL, respectively [Fig. 3(3), 3(4) and 3(5)]. On an average, *Legionella* spp. could be detected at concentrations of as low as 13 CFU/100 mL after filtering and culture enrichment.

### Test of real water samples and its confirmation by sequencing

Seven samples of condensed water from air conditioners collected and provided by the Center for Disease Control and Prevention, Shanghai, China, were subjected to the microarray analysis. The hybridization profiles of the samples revealed that three of the seven samples were contaminated by *L. pneumophila* [Fig. 3(1)]. The remaining four samples generated signals with the positive control probe indicating the existence of bacteria other than the ten *Legionella* spp. (Fig. S3). The existence of *L. pneumophila* in the three water samples was confirmed by PCR amplification and DNA sequencing of the *L. pneumophila wzt* genes (23).

## Discussion

Bacterial species have at least one copy of the 16S rRNA gene and the 16S-23S rDNA ITS region contains both highly conserved regions and hyper variable regions, which are useful molecular markers for bacterial identification at the species [26] and subspecies levels [27], [28], typing [29], [30], as well as in evolutionary studies [31], [32]. In this report, we describe a microarray method for the determination of pathogenic and non-pathogenic *Legionella* spp. on the basis of their ITS regions.

A number of techniques have been adopted to improve the reproducibility and sensitivity of the microarray First, we used a two-step PCR to amplify and label the samples. At the first step, the forward and reverse primers were used to amplify the target genes; followed by the labeling of single–strand DNA using the reverse primers at the subsequent step. The two-step PCR scheme enhances the amplification efficacy and generated intensively labeled probes as well.

The microarray is sensitive, and as little as 1.0 ng DNA or 13 CFU/100 mL can be reliably detected. This is achieved using a two-step procedure of vacuum filtering and culture process to enrich the target *Legionella*. After the collection of all the bacteria in the samples by the vacuum filtering, the acid-resistant *Legionella* were treated with HCl and selected in BCYE or GVPC [17]. The process described here allowed the detection of *Legionella* in a short time of 2–3 days, which is 9–10 day faster than the existing methods of identification (ISO11731∶1998).

The three probes OA-3815, OA-3816, and OA-3817 used to identify *L. pneumophila* are species-specific rather than subspecies-specific. Of the 18 strains of *L. pneumophila* obtained (Table 1), 14 were *L. pneumophila* subspp. *pneumophila*; three were *L. pneumophila* subspp. *fraseri*; and one was *L. pneumophila* subspp. *pascullei*. All the 18 strains were identified as *L. pneumophila* by the array. Only one or two reference strains were used for non-*pneumophila Legionella* species (Table 1) due to the limited availability of strains of these species in public culture collections.

In both ITS-tRNA^Ala^ and ITS-tRNA^Ile^ phylogenetic trees, the three *L. pneumophila* subspp., namely, *pneumophila*, *fraseri*, and *pascullei,* were grouped together as expected. *L. pneumophila* was found to be phylogenetically close to *L. fairfieldensis* but distant from *L. longbeachae*, while *L micdadei* was found to be in the neighborhood of these two species. As reported, most (approximately 90%) of the Legionnaire disease are caused by *L. pneumophila*, and of the 15* L. pneumophila* serogroups identified, O1 alone is responsible for more than 84% of the cases of Legionnaire’s diseases worldwide [33], [34], [35]. Other Legionnaire’s diseases were caused by two common pathogenic *L micdadei* serogroups. The phenotypic characteristics of *L. micdadei* were reported to be quite similar to that of *L. pneumophila* and *L. bozemanii* [36]. *L. longbeachae* is another causative agent of Legionnaire’s diseases in Australia and New Zealand, and is associated with exposure to potting soil [37]. *L. fairfieldensis* was isolated from water in a cooling tower in Fairfield, Victoria, Australia, in February 1987, but has not yet been recognized as a human pathogen [6]. Although *L. fairfieldensis* is nonpathogenic, its ITS sequence was most closely related to *L. pneumophila* (the identity of 16S rRNA sequences between *L. pneumophila* and *L. fairfieldensis* is 0.909, while the identity of *mip* genes of these two is 0.587). A comparative study of *L. pneumophila* and *L. fairfieldensis* genomes could provide further insight into the pathogenesis of these bacteria.

## Conclusion

We have sequenced and analyzed the ITS regions of 12 *Legionella* spp., *L. anisa, L. bozemanii, L. dumoffii, L. fairfieldensis, L. gormanii, L. jordanis, L. longbeachae, L. maceachernii, L. micdadei,* and *L. pneumophila* (including subspp. *pneumophila*, subspp. *fraseri*, and subspp. *pascullei*). We found that only *L. jordanis* contained three distinct ITS types: ITS-tRNA^None^ (without a tRNA gene), ITS-tRNA^Ala^ (with tRNA^Ala^ gene) and ITS-tRNA^Ile^ (with tRNA^Ile^ gene), and the rest nine *Legionella* spp. contained two distinct ITS types: ITS-tRNA^Ala^ (with tRNA^Ala^ gene) and ITS-tRNA^Ile^ (with tRNA^Ile^ gene). Alignments of the above ITS sequences revealed significant interspecies variations of 0.266–0.772 for ITS-tRNA^Ala^ and 0.280-0.774 for ITS-tRNA^Ile^, but low intraspecies polymorphisms of 0.967–0.993 for *L. pneumophila* ITS-tRNA^Ala^ and 0.943–0.996 for *L. pneumophila* ITS-tRNAI^le^, which provides a molecular basis for species-specific identification.

We have developed a rapid oligonucleotide microarray to identify the above 12 *Legionella* spp. and subspp. based on the polymorphism of 16–23S rRNA ITS sequences. A total of 52 strains were used to test the microarray assay, including 30 target pathogens and 22 closely-related bacteria. The 29 probes selected have reproducibly detected multiple pathogens with high specificity and sensitivity at 1.0 ng genomic DNA or 13 CFU/100 mL following filtering and culture enrichment. A 100% detection of the seven air conditioner-condensed water samples validated the microarray. Our findings revealed that the oligonucleotide microarray technique presented in this study is a promising method for basic microbiology, clinical diagnosis, food safety, and epidemiological surveillance.

In conclusion, this study presents a new PCR-based microarray assay for the comprehensive and simultaneous detection and identification of ten *Legionella* spp. This new method provides an accurate and reliable approach to differentiate among *Legionella* isolates at the species level; contributes significantly to large-scale epidemiology studies; and can be used to monitor local, regional, and national trends in human legionellosis.

## Supporting Information

Figure S1 **Agarose gel electrophoresis of PCR products of** ***Legionella*** **spp. ITS regions.** Lanes: M, molecular weight standards (DL2000 Marker); (1) *L. anisa*; (2) *L. bozemanii*; (3) *L. dumoffii*; (4) *L. fairfieldensis*; (5) *L. gormanii*; (6) *L. jordanis*; (7) *L. longbeachae*; (8) *L. maceachernii*; (9) *L. micdadei*; and (10) *L. pneumophila*.
(TIF)

Figure S2 **The sensitivity of the microarray analysis with genomic DNA of** ***L. dumoffii*** **.** (1) 100 ng; (2) 10 ng; (3) 1.0 ng; and (4) 0.1 ng.
(TIF)

Figure S3 **Microarray pattern of bacteria other than the ten** ***Legionella*** **spp.**
(TIF)
