# Supplementary figures and images for: A New Oligonucleotide Microarray for Detection of Pathogenic and Non-Pathogenic Legionella spp
Source: PLoS One. 2014 Dec 3;9(12):e113863. doi: 10.1371/journal.pone.0113863 (PMC4254607; doi:10.1371/journal.pone.0113863)

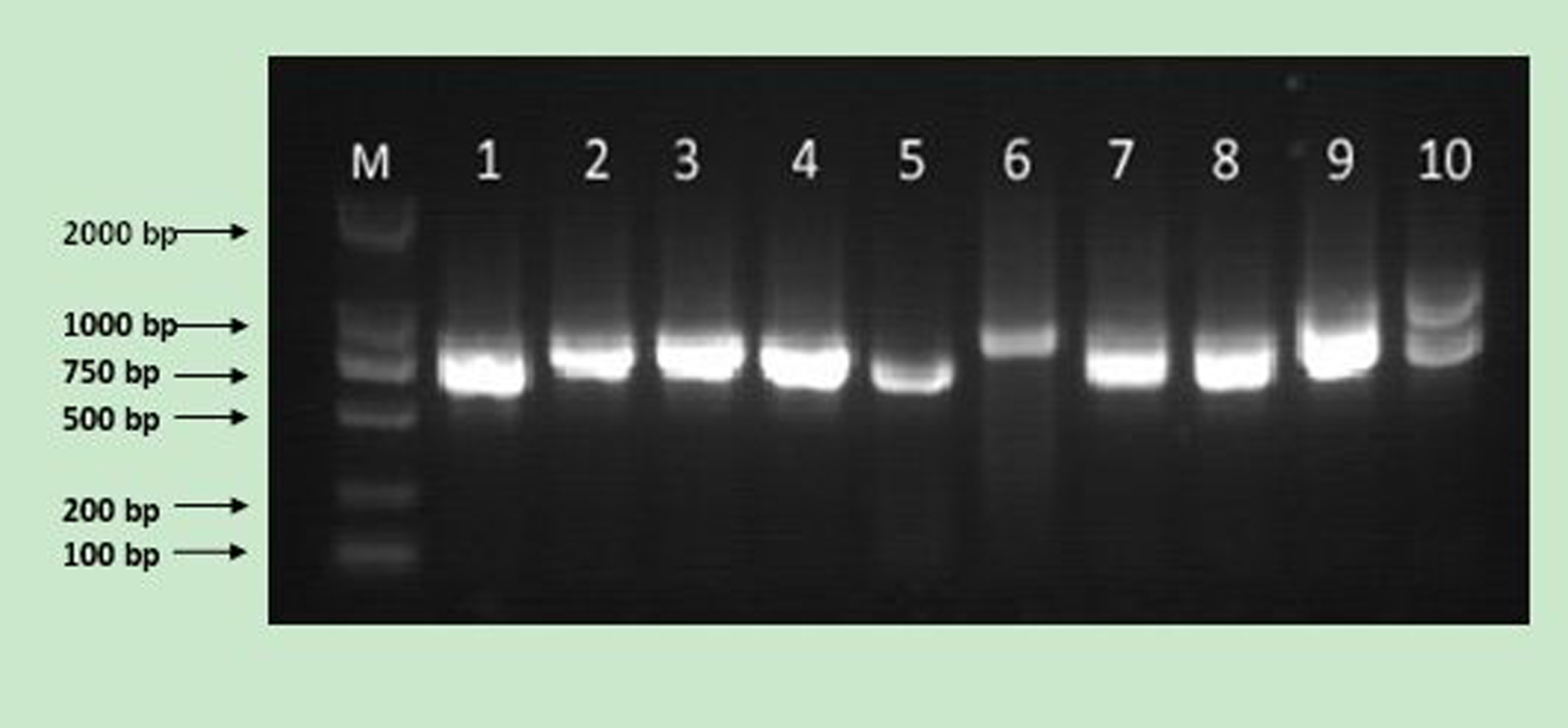

Supplement: Figure S1 — Agarose gel electrophoresis of PCR products of Legionella spp. ITS regions. Lanes: M, molecular weight standards (DL2000 Marker); (1) L. anisa; (2) L. bozemanii; (3) L. dumoffii; (4) L. fairfieldensis; (5) L. gormanii; (6) L. jordanis; (7) L. longbeachae; (8) L. maceachernii; (9) L. micdadei; and (10) L. pneumophila. (TIF) [file pone.0113863.s001.tif]

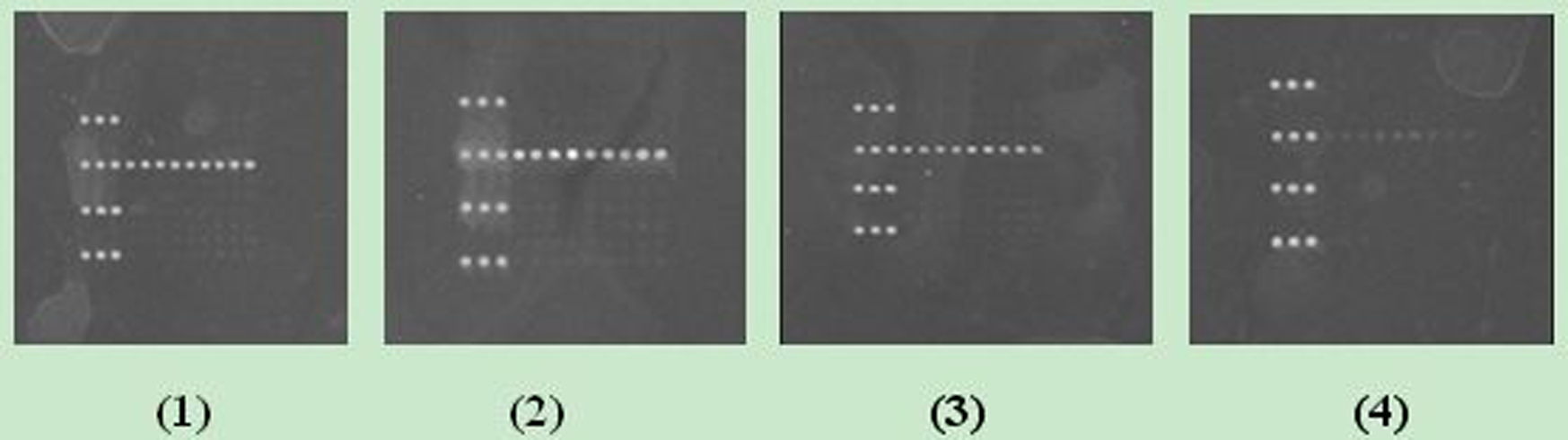

Supplement: Figure S2 — The sensitivity of the microarray analysis with genomic DNA of L. dumoffii . (1) 100 ng; (2) 10 ng; (3) 1.0 ng; and (4) 0.1 ng. (TIF) [file pone.0113863.s002.tif]

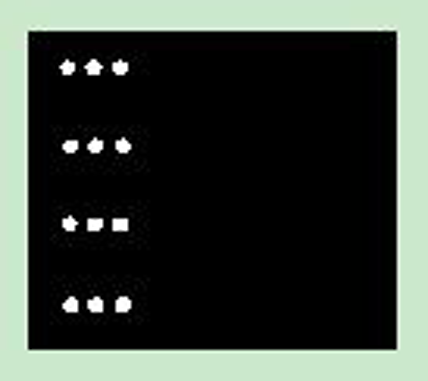

Supplement: Figure S3 — Microarray pattern of bacteria other than the ten Legionella spp. (TIF) [file pone.0113863.s003.tif]
